# Digital Storytelling as a Reflective Tool in Occupational Therapy Curriculum

**DOI:** 10.1155/2021/2463916

**Published:** 2021-09-16

**Authors:** Amshuda Sonday

**Affiliations:** Division of Occupational Therapy, Department of Health and Rehabilitation Sciences, Faculty of Health Sciences, University of Cape Town, South Africa

## Abstract

With a shift in moving towards the 4^th^ industrial revolution, digital storytelling has been identified as a novel way of facilitating teaching and learning. This paper will be aimed at offering an understanding of the experience and perspective of occupational therapy students in using digital storytelling as a reflective tool as an assignment as part of their undergraduate and masters occupational therapy curriculum at a university in South Africa. A descriptive qualitative study was undertaken, and five participants were purposively recruited. Individual semistructured interviews were conducted as well as a focus group with participants. An inductive analysis revealed two themes: *Reflections on relevance within the occupational therapy curriculum* and *Is technology the new direction?* The findings conclude that digital storytelling as a medium to showcase reflections on identifying formation was an innovative and novel way of documenting the reflective experiences of occupational therapy students.

## 1. Introduction

Digital storytelling is a concept that was created in the late 1980s by Joe Lambert and Dana Atchley who were the cofounders of the Center for Digital Storytelling (CDS). According to Lisenbee and Ford [1], digital storytelling is defined as making use of various technological tools, such as an interactive whiteboard (SMARTboard), computer, cellular phone, or tablet to share narratives, images, and experiences in a multimedia form. Digital storytelling can be used to combine personal experiences and meaningful content based on the context individuals find themselves in. Digital storytelling in the learning context can harness critical thinking and problem solving skills, collaboration with others, and opportunities to allow the students to use their personal creativity and theoretical knowledge to narrate their own experiences [1].

Digital storytelling has been increasingly incorporated into the educational system by educators in the Global North defined by [2] as societies and countries such as Europe and North America are developed in terms of democracy, technology, wealth, and politics. The incorporation of technology into learning was to try and encourage autonomy over learning and spark interest via the use of technology [1, 3]. Skarpaas et al. [4] in Norway used digital storytelling to explore poetic reflection in OT. The purpose of LeFrance and Blizzard [5] study was to examine and explore the perceptions students have of storytelling as a pedagogical tool to allow engagement in meaningful self-reflection and understanding the theories through the process of reflection. Skarpaas et al. [4] examined the contribution of digital storytelling to OT students' learning through reflection on their experiences from their placement education. Students identified reflection as one of the important outcomes of using digital storytelling as a reflective tool and enjoyed the process of creating and sharing their digital story. Young people today are influenced by media and find digital activities such as blogs and wikis more appealing, and so by using these mediums, they can use their own experiences and creativity to make sense of everyday life themes and issues [3]. Lisenbee and Ford [1] stated that digital storytelling gives students the opportunity to make authentic connections between their subjective meaningful experiences and academic content to enrich learning and understanding. However, there is limited literature that explores the use of digital storytelling and the experiences of individuals after they have engaged in the process, especially within the South African context. This led to the research question *How have occupational therapy (OT) students experienced the use of digital storytelling as used in the undergraduate and master's occupational therapy programs at University of Cape Town (UCT)?* This paper will be therefore aimed at offering an understanding of the experience and perspective of occupational therapy students (undergraduate and masters) in using digital storytelling as a reflective tool as part of an assignment linked to their respective OT courses at a university in South Africa.

## 2. South African Perspectives on Digital Technology in Education

In 2004, the former Minister of Education, Naledi Pandor, noted that “Digital media has revolutionised the information society and advances in Information and Communications Technologies (ICTs) have dramatically changed the learning and teaching process” ([6] as cited in [7], p. 1). The White Paper 7 on e-Education [6] describes a global revolution that is currently happening in education and training, primarily driven by the changes evident in the type of work available, living in the digital age, a need to keep up with the developed Global North, and the need for equal distribution of resources in education.

A recent study conducted by Chikuni and Chigona [8] looked at the e-learning policies within a university in South Africa to reflect on the policy discourse around e-learning and technology integration in Higher Education Institutions (HEIs). The aim of the study was to compare the pros and cons of an e-learning institutional policy while situating it in its sociopolitical context [8]. The e-learning policy from a South African University of Technology (UoT) was analysed. The findings revealed that the institutional policy of the UoT can be considered technocratic policy as it is said to create the illusion that technology is being successfully integrated into the practice of education. However, it does not take into account the pedagogical issues that are inherent to the practice of teaching and learning [8].

The challenges of using technology within education are evident when examining the context which we are in. According to the General Household Survey [9], 59.3% of South African households reported having access to the Internet either at work, school, home, or an Internet café while only 9.3% reported having Internet access readily available in their homes. The College of Health Sciences (CHS) at the University of KwaZulu-Natal implemented a visual learning project giving 1400 of its student's access to Proline Tablets. This technology allowed students to livestream their lectures and record lectures to watch later on. During the livestreamed lectures, students were also able to ask their lecturers questions electronically while in the lecture hall or at a remote location [10]. Findings from the study relates to the idea that students want to be able to access information conveniently choosing their own time and location to learn; however, a big issue is still the accessibility of Wi-Fi and connectivity issues off campus at home in rural areas compared to urban settings [10].

## 3. Reflection as a Tool to Shape Professional Thinking

Reflective learning is known to encourage the development of critical thinking, self-awareness, and analytical skills. Being a reflective learner generates meaning and purpose in the learning experience [5]. Fenwick [11] as cited by LaFrance and Blizzard [5] posits that an individual is able to learn from a concrete experience through a reflective process that needs to happen separate from the experience. While looking at the perceptions of students after the use of digital storytelling as a learning tool for educational leaders, LaFrance and Blizzard [5] reported that the primary benefit noted by students was the opportunity to reflect, being able to take the time to reflect back on their past experiences. Students reported that the digital storytelling had given them the space and capacity to reflect on their experience in a way that a written assignment would not have been able to allow. The level of engagement required to do a digital story is quite high; therefore, retaining that information learnt through using digital storytelling became easier [5]. The use of digital storytelling also exposed students to different technology and software applications; for some, this was seen as a learning opportunity while others felt it was an aspect they found particularly challenging to navigate [5]. A challenging aspect that came up in this study was related to having to share personal information in the digital story; students found it difficult to get into the topic of the assignment while avoiding addressing situations they found humiliating or embarrassing [5].

### 3.1. The Digital Story Assignment

The digital story assignment was introduced as a pilot at the third year level and was thereafter adapted and used as part of a master's course in occupational therapy. The purpose of the assignment was for the students to create a digital story using reflections on experiences of their professional identity development across a 10-week physical health practice learning setting. These 10 weeks were structured in two 5-week blocks. This assignment was introduced in a lecture format. The process was outlined to the students as the following:

#### 3.1.1. Preparation before the Start of Practice Learning

An introductory session was conducted to the class outlining the expectations of the assignment and providing more information on the definition of digital storytelling and professional identity. Training on how to create a digital story was provided by a Health Sciences Education expert on technology within the classroom. Additional resources on how to use the various technological mediums were uploaded onto a central electronic site where all students had access to.

#### 3.1.2. During Practice Learning

The students needed to identify critical incidents related to professional identity while they were on their fieldwork. Collection of digital images, music, picture, etc. during the practice fieldwork would help support or enhance their digital story. As part of the third year practice learning curriculum, students were expected to document learners' logs, which are weekly reflective narratives of their experiences.

#### 3.1.3. Process of Creating the Digital Story

The digital story had to include information about how the student felt at the beginning of the placement. Students were encouraged to make notes on their first responses to the placement (learners' log in week 1). The story had to be informed by the learning objectives of the assignment. Students were expected to reflect on action on the development of their identity as an occupational therapist. Mapping out their 10-week trajectory served as an outline to their digital story.

The works of Sandars et al. [12] and Ivala et al. [13] were used as a guideline for students to map out their trajectory over the 10 weeks:

- Decide on a topic/focus for the story (tutorial in week 1 of the 2nd block). The students had to identify one incident or situation that occurred in the 10 weeks, where they had to negotiate their professional identity
- Share the story with peers in the tutorial group
- Write the story—this will be a brief script (story board) giving the structure to the story
- Collect a variety of multimedia to create the story (throughout block 2)
- Select the collected multimedia to create the story
- Create the story using chosen technology
- Submit the digital story on Vula, which is the University of Cape Town online collaboration and learning system

### 3.2. Evaluation of the Digital Story

An assessment rubric modified from Barrett [14] was used to evaluate the digital stories. The rubric incorporated the choice of topic, use of media, and new insights gained [12]. The digital stories contributed 5% towards the formative evaluation for the Physical Health in Occupational Therapy course (AHS3107W).

## 4. Methodology

A qualitative descriptive method was chosen in order to understand and explore the beliefs, experiences, attitudes, behaviour, and interactions [15] of the participants in the study. The study was conducted by six final year occupational therapy students as part of their research project. Participants were purposively [16] selected from UCT's Division of Occupational Therapy based on the following selection criteria: students who must be within the Division of OT at the Faculty of Health Sciences (UCT) and students who have used digital storytelling as a reflective tool for one of the courses offered within the OT curriculum and can either be third year or master's OT students. Data was collected by the researchers, using semistructured interviews [17] which were 60 min in duration. A focus group was held and facilitated by the researchers as a form of consolidation and member checking to ensure that the participants' experiences had been captured and interpreted appropriately. An inductive approach was taken to analysis; using thematic analysis, the researchers analysed the text derived from the data collected (Braun & Clark as cited in [18]). Through the thematic analysis process, patterns or themes within the research data were identified. This process consisted of transcribing all recorded material and coding the data by identifying units of meaning and naming the units. It was then followed by developing categories through establishing relationships and patterns between the codes and generating themes. Saturation was achieved once there were no new themes, no new codes, and no new data emerging (Fusch & Ness [19]). Since the researchers were OT students themselves, bracketing [20] was used during engagement with the data and with the evolving findings that emerged in the focus group. The researchers identified and temporarily set aside assumptions they had with regard to the research topic. Secondly, the researchers revised the data findings as well as their evolving comprehension of it so that they could achieve a revised understanding of any aspect of the research topic and findings [20]. This was done in consultation with the research supervisor.

The ethical approval from the Faculty of Health Sciences Human Research Ethics Committee (HREC REF: 2018/0840) and Department of Student Affairs at the UCT was obtained.

## 5. Findings

Two themes and corresponding categories emerged. The first theme *Reflections on relevance within the OT curriculum* and the second theme *Is technology the new direction?* address the process and emotional impact experienced by the participants during the use of digital storytelling.

### 5.1. Theme: Reflections on Relevance within the OT Curriculum

This theme describes the thoughts and experiences of the OT students in understanding the assessment process and how this new way of learning could potentially facilitate learning and describe the perceived barriers and benefits of using digital storytelling in higher education learning environment.

#### 5.1.1. Digital Storytelling as an Assessment Tool

The participants reported that they did not understand the objectives of the instruction due to being too broad and vague. They expressed that the rubric lacked structure and was subjective and not standardized.

*The objective and the rubric, it was very vague and very like it was just too broad. [Participant 4]*

*We were marked on it, hated that, because of its subjectivity [Participant 3]*

All of the participants found the use of digital storytelling as an assessment tool to be unfair. Participants expressed that the marks they received did not reflect the effort and work that they had put into the assessment.

*I know like, some people put like, real effort and their things looked really nice and they got like average marks. [Participant 4]*

*I think the subjectivity of it shouldn't have a mark attached to it ‘cause then it's saying your experience isn't valid, your reflection isn't valid [Participant 3]*

Suggestions were made on how the rubric could be restructured to reflect a more standardized and fair assessment. It was suggested that majority of the marks should be weighed more on the content of the digital story rather than on the presentation of it. Other participants felt that a mark should not be attached to digital storytelling at all because of its subjectivity and to avoid bias.

*A very subjective tool and they'd have to really fine tune the … the criteria of it and what they're expecting from students if they're gonna be using digital storytelling as a reflective tool. [Participant 3]*

*Majority of the weighting would have to be about the content and not actually looking at the presentation. [Participant 5]*

#### 5.1.2. Facilitating Learning

The participants were asked if the use of a technological medium facilitated their learning experience. The use of a digital story allowed them to be more involved and invested in their learning process as it solidified their learning and made the learning journey clear and more real.

*Putting it into pictures kind of made it clear for me … my learning journey. [Participant 4]*

*It definitely helped with my learning and solidifying what I had already come to know. [Participant 3]*

*I don't really relate, I think I just knew the structure for me to learn from it. [Participant 1]*

During the focus group, the participants spoke of how digital storytelling facilitated their learning. The participants mentioned that digital storytelling helps them to reflect better compared to their written narrative reflection, using images and music assisted greatly in showcasing their thoughts in cases where words would not do it justice. The development of their technological skills was also enhanced through doing this assignment, and it also helped them to reflect on their emerging capabilities:

*I felt like I really reflected on my emerging capabilities as an Occupational Therapist. [Participant 4]*

*Helped within your own reflection. [Participant 1]*

*It as a growing experience, growing not only your skills with technology but then also reflecting in a different way. [Participant 2]*

*Good reflecting space...creating something but also creating the opportunity to think back on an incident. [Participant 1]*

A discussion emerged between the participants about how digital storytelling can be further used in the OT curriculum to facilitate learning and what the benefits of it would be. One participant had the opportunity to share her digital story with her classmates, and they did the same. The participant shared how this was a benefit to their learning as it provided them the opportunity to learn from each other. The participant also mentioned how this way of learning kept everyone engaged.

*It's nice as a per tool but I would have liked it to be kind in a collaborative way within the class as well… you could have learned from someone else's digital storytelling. (Participant 3)*

*It helps people to invest in their learning because you are teaching your peers and your peers are teaching you, and that's how we as adults are supposed to learn. (Participant 5)*

#### 5.1.3. Perceived Benefits and Barriers

The relevance of the digital means in relation to the technological era we are in was apparent through the interviews. One of the participants mentioned that incorporating digital means into the curriculum ensures that we are “staying relevant, keeping people interested, different learners, more inclusive” (Participant 2). In contrast, some participants voiced that they do not prefer technology, but they agree that it needs to be incorporated into the curriculum because the world is quickly turning everything into digital material (Participant 4).

*I don't want to agree with it but I have to agree with it because we are in the fourth industrial revolution… I don't like using technology… the world is moving towards technology [Participant 4]*

*Incorporate new things into the curriculum to keep up with the time [Participant 5]*

Clear benefits that the participants identified were the creative means of conveying experiences and knowledge. It was an added benefit when it is used in a learning space. One participant stated that it allows the students to learn from each other, “validate or appreciates the knowledge of the students at the same level as a lecturer” (Participant 5). It was also voiced that if digital storytelling was used in the curriculum, it would make the learning material more accessible.

*It would be much better to learn from [Participant 1]*

*Easier way of absorbing the content [Participant 3]*

*It forces us to dig deeper into one or more experiences, more than you might go into a learner's or a reflective log [Participant 2]*

It was clear that the participants saw benefit in the new medium, but there are still a few barriers that need to be overcome.

*Having different medium of being taught, can really spark interest [Participant 3]*

*They will need to do fine combing but I still think it should be done [Participant 2]*

The biggest barrier that was identified spoke to how digital storytelling cannot be used as a mainstream reflection tool because not all contexts allow the student to use the tool. Even when we are in an era that is moving in the digital era, not all people/students have access to technology in order to create a digital story.

*It's context related… it won't be something that can be a mainstream tool. [Participant 3]*

A participant stated that they did not see more growth when using the digital story in comparison to other reflective tools.

*It does not make a difference to writing a learner's log or reflective log [Participant 1]*

*It would have been the same effect [Participant 1]*

Another participant went on to say that they would have preferred to do a writing piece and do a learner's log instead.

*Having to put on a PowerPoint for people to see would be a bit exposing [Participant 5]*

*There will be a clear difference between what she would share in a written log versus a digital story [Participant 5]*

And lastly, participants also said that because it requires more effort than writing a log, they were more focused on other academic requirements.

*I kind of saw it as just this like added extra thing [Participant 2]*

*I for one like writing. It would definitely be a different process and what I would choose to share would be very different [Participant 5]*

Participants spoke about new beneficial possibilities because in their experience, some participants found that digital storytelling created a positive space for them to reflect. One of the benefits included that it would increase the awareness that lecturers will have around what happens at the practice learning sites.

*It would give like our lecturers a bit more of a glimpse on what we go through in block [Participant 4]*

One of the participants had the opportunity to be part of the learning space where the students had to share their digital story with the rest of the class. After they had created their digital stories about their experiences, each student had the opportunity to play the digital story to the class and she identified benefits that she experienced while this happened.

*It removed performance anxiety [Participant 5]*

*It made the lecture space more exciting [Participant 5]*

The participants acknowledged that there are benefits as well as barriers that digital storytelling pose to the OT curriculum. Some stated that they were more interested in engaging in the reflective tool because it allowed them to engage with technology, while another stated that she would not share as much of her experience as she would when writing because she feels exposed when doing a digital story. When weighing the benefits and barriers, the participant stated that if the tool is used to reflect, learn, and teach, then it would be very beneficial.

*Engagement with technology made them more interested in engaging [Participant 2]*

*If it was purely for reflection then it would take too long but if it was teaching, learning and reflecting all in one, i think it would be very beneficial [Participant 2]*

*One beautiful thing about OT is that there is no one right way to achieve a goal. As long as it's used meaningfully [Participant 5]*

#### 5.1.4. Broadening Digital Storytelling to Different Clusters

The Division of Occupational Therapy at UCT is structured conceptually according to varying domains of practice, which are termed clusters. There are five clusters (physical health, mental health, work practice, child learning, development and play, and community development practice) that are made up of staff members whose expertise lies within that domain of practice. Relevant to this study is the physical health cluster. Participants stated that “the quality of your reflection very much depend on where your block was” (Participant 2). The participants spoke about how they would have wanted their digital story experience to have been open to more clusters within the OT curriculum. Some were happy with the cluster that their digital story reflection was based on:

*The physical block I had was quite an enriching experience for me so it was very easy to get an idea for the digital storytelling. [Participant 3]*

*I did mine on an experience in my 4th block so I had gone through three other blocks that I had to hand reflective logs [Participant 2]*

Theme 1 offered some insights into the benefits as well as barriers that emerged that participants shared about using this digital tool that speaks to how moving into a fourth technological era affects the world. Benefits included how the tool incorporates creativity with the curriculum and barriers spoke to how making use of technology is very context-based and that limited access or quality means that it cannot be used as a mainstream tool. All participants experience digital storytelling as an assignment assigned to a specific area of practice learning. Participants stated that it would have helped to broaden their digital storytelling experience to practice learning clusters.

### 5.2. Theme 2: Is Technology the New Direction?

This theme describes the influence that technology plays within teaching and learning. Additionally, it will also describe the understanding from the participants' perspective, a new direction that is being paved in education, with technology being used as a teaching and a learning tool.

#### 5.2.1. The Process and Emotional Impact

The steps of creating a digital story for fourth year OT students included using a technology medium and identifying an experience in which they wished to reflect upon from their physical health practice learning block (hereafter block). In order to produce a digital story, participants shared that they first had to complete their block and then they had to find an experience which they wanted to share and reflect upon. Even though participants had received this instruction, there was still confusion on the expectations of the assignment. Participants had to refamiliarize themselves with the objectives so that they could start the process of creating their digital story.

*I did the blocks and then read the brief again and then had to think back about everything that happened [Participant 2]*

*It forced me to first of all find and experience and then I read the brief again then and experience came to mind that I wanted to reflect [Participant 2]*

Once participants were clear on the experience which they wished to reflect upon, they had to use a technology medium to create their digital story. There was a mix of technology mediums used to create a digital story among the fourth year participants. For those who used PowerPoint, they had to share their experience using voiceover on PowerPoint; others used a movie program to create their digital story.

*Having to record little bits for each slide...then with the whole transitioning from one slide to another [Participant 5]*

*I didn't use PowerPoint presentation...I used uhm...a movie making app program… [Participant 2]*

*I had to use PowerPoint [Participant 1]*

*I also did something else besides a presentation. I did like a musical montage [Participant 3]*

When it came to the process of making a digital story, one participant shared that owing to the fact that the objectives were broad and unclear, it allowed them more independence and allowed them room to interpret the assignment the way in which they had understood it. This provided them with room to be more creative with the way in which they wished to present the assignment and the content that they wished to include in it.

*You could really take it wherever you wanna go [Participant 3]*

*I had a lot of independence on how I got to convey my learnings” [Participant 3]*

For the master's student, the participant was required to present a narrative on the critique of physical health practice in OT. The participant shared a similar experience of the fourth year participants who had to use PowerPoint to create their digital story. Once they had created their digital story, the participant had to share their digital story with their peers.

*I kinda struggled with it...PowerPoint did not play ball with me...cause you know you have to record your voice for each slide but my recordings were not compatible with that version of PowerPoint [Participant 5]*

Participants articulated that there was enough time to complete the assignment; however, there were other factors related to time which impacted the participant's engagement with digital storytelling. Participants shared that the timing of when the assignment was given was not appropriate and the creation of the digital story took more time than expected. Other participants reported that, in the third year of their OT program (when most of the participants were first introduced to the tool), they were feeling overwhelmed and having to engage with the new tool took a lot out of them. Another participant shared that it was during the time when the faculty was going through the loss of its dean. The emotional climate at the time filtered on their engagement with the tool.

*They definitely gave us sufficient time [Participant 5]*

*The timing of it was just horrible [Participant 4]*

*It was time consuming [Participant 5]*

As a result of poor timing and other time-related issues raised by participants on the creation of a digital story, fourth year participants shed more light on the emotional climate around the time digital story was introduced to them. Participants echoed that they were overwhelmed, and having to engage with a new tool for reflection “takes a lot out of you as well” (Participant 1). One participant shared the different emotional influences such as the death of the Dean of Health Sciences which negatively impacted their engagement with digital storytelling.

*It was also around the time of Prof Mayosi's passing… that influenced my level of engagement in the actual digital storytelling [Participant 5]*

Another participant shared their experience with being vulnerable about their experience while creating their digital story. Participants echoed this sentiment as being vulnerable puts an emotional weight on the person sharing, and this was intensified as the participants were uncertain with how their emotions would be received by markers.

*It's such an emotional taxing thing to do; to be able to open yourself up to - sometimes lectures being insensitive [Participant 4]*

During interviews and a focus group, participants shared their reflections on their process of creating a digital story. There was a mixture of reflections with each participant sharing what they felt about the process which they underwent.

*I wasn't interested in it...so I didn't put much effort into it [Participant 1]*

*It was more about like getting it done [Participant 4]*

*I again liked the creative process behind it [Participant 3]*

#### 5.2.2. Technological Capacity

Most of the participants expressed that the use of digital storytelling on a larger scale would be difficult, citing factors like access to the Internet and a technological device appropriate to complete the project as commodities that they foresee being inaccessible to many people. Participants noted that access to different technological equipment was a contributing factor that could limit a person's participation. Some participants commented on the possible discrepancy that could be evident in the final product as a result of people having access to different kinds of equipment at their disposal.

*Access to those digital things like for example like using phones, cause some people could have used a camera to film [Participant 3]*

Another point highlighted by many participants was the inability to access other types of software applications that could have been used for creating a digital story. There was consensus among participants that using PowerPoint did not allow them the desired creative range, describing it as time-consuming. One participant who was the only person with access to a software application other than PowerPoint reported that she had a great deal of fun making a digital story because she used a movie making program on her computer that allowed her to create a stop-start promotion animation video, but she felt that if she had to go through the process using PowerPoint, she would have found it tedious.

*My recordings were not compatible with that version of PowerPoint so it was a bit of a mess [Participant 5]*

*I had to use PowerPoint because I didn't know how to use anything else. So that was a bit tedious, I think [Participant 3]*

Previous technological knowledge and skills were mentioned by the participants as factors that were beneficial. Some participants were excited that there was a digital element to the task that they had to complete, citing high school exposure to technology as having put them in a fortunate position to understand and execute the task well. Other participants reflected on the difficulty of the level of skill in terms of technology that is required to be able to create a digital story noting that it would be challenging if they had not had experience with technology prior to completing this task.

*Although our technical and computer skills do grow over the course of our learning period of like university career its- it may be a bit too hard considering that we all come in with the different computer skills [Participant 5]*

*I don't know a lot about technology [Participant 3]*

It came up from the individual interviews that participants also found it challenging to operate the technology software applications they had access to, and this combined with their confidence in their technological abilities made the process an unpleasant experience sometimes. This compounded with the time crunch others were under leads to feelings of frustration.

*I did not like using technology it was so hard, it makes you feel so stupid because like when you don't know how to put voice overs on PowerPoint, and like sometimes when you recording it's unclear so you have to do it again. It's very frustrating… [Participant 4]*

These experiences were juxtaposed by two other participant experiences; these participants described their enjoyment for the digital nature of the task acknowledging that they have always been tech savvy. This task allowed them the ability to play and be creative while still facilitating a learning experience.

*I liked the digital aspect of it so I was able to edit, I was able to do all that [Participant 3]*

#### 5.2.3. Learner Inclusivity and Mixed Experiences

The participants relayed their opinions on how they experienced/would experience the tool in teaching spaces. The master's student was the only one who had this experience while the undergraduate students were making assumptions.

*So having digital storytelling as a medium of teaching would be exciting for me, I'd actually pay more attention [Participant 5]*

Some of the participants described themselves as visual learners, acknowledging that having the opportunity to make a digital story was a different experience that afforded them the opportunity to learn and experience a different way of reflecting. Reflecting in this manner was also described by one participant as being an inclusive way to learn that it accommodated the different types of learners within the class.

*I think the fact that we are afforded different tools to reflect is really nice because we have the learners log and then we have the DS and that's… It's kinda' two of the same but it reinforces your emergence as a competent OT. [Participant 3]*

Participants expressed their enjoyment of the use of a different learning format other than the traditional model of attending lectures or reading and writing. Digital storytelling-incorporated technology into their learning in a new way that participants felt would better the way people who learn differently would be able to appreciate and benefit from. Most of the participants said they were visual learners or enjoyed the incorporation of creative elements within their learning, and for them, digital storytelling encompassed all of these aspects that facilitated their learning.

*So like having a different medium of being taught, can really like spark interest cause I know that me I'm a visual learner I like seeing things, I like you know grappling with content in that way. [Participant 3]*

*I don't do well with like writing and reading. I like looking at pictures I like, like watching, like YouTube videos to learn. [Participant 4]*

A common feeling among some of the participants was that they were not technologically inclined people who were confident in their technology skills and that it affected how they engaged with the task. Sharing that because of their limited familiarity with PowerPoint, the experience was less enjoyable for them in the beginning because they did not feel that they had adequate skills to complete the task. Two participants felt that the structuring of the task was great because of the inclusion of technology and that it increased their interest in completing the task.

*I think at first it was scary because I'm not really like a technological person, I'm not really good with like being creative [Participant 4]*

It also came up in some of the individual interviews that participants self-identified as not being inherently creative reporting that they would have preferred to physically write the task than create a digital story. For some, making the digital story took more time than writing a reflective log would have and required more effort with participants saying they would have preferred to write than to make a digital story. This is contrasted by participants who enjoyed the shift from the usual reflective modes used, appreciating the creative ability they were able to express and show off in the creation of a digital story.

*I would still prefer to write ‘cause it's quicker [Participant 5]*

*I kinda enjoyed making something rather than writing out an essay [Participant 1]*

A participant who had to present their digital story in front of their class shared that they thought creating a digital story and presenting a PowerPoint in that way made it less nerve wracking and inclusive for people who may find it difficult to speak in front of crowds and present. According to the participant, this in turn changed the learning space within that class, encouraging peers to share and teach each other viewing the learners as adult learners who have their own knowledge and experiences to bring to the table.

*It gives you the opportunity not to have stage-fright [Participant 5]*

Participants' experiences on the whole experience of using digital storytelling and on what they took out of the process also varied. It was felt that it was a very subjective, reflective experience that allowed their creative side to come out. Participants also reported on the growth that came with engaging with the tool, and for some, the growth only came after they had received their feedback.

## 6. Discussion

Understanding the shift in focus within OT practice and education calls for a deconstruction of the OT curriculum for both undergraduate and postgraduate programs. This shift explores how digital storytelling situates itself within the OT curriculum with the intent to highlight the integration of technology within education.

### 6.1. Shift in OT Practice and Education

From the findings, some of the participants stated that digital storytelling aided their reflective process, and thus, it can be agreed that reflection and digital storytelling are important within occupational therapy programs. It was also acknowledged that this might not be applicable to all the students, as others found that the digital means did not aid their reflective process. While acknowledging that reflection is a critical element in the making of an occupational therapist, it is important to note that it was found that in this study, digital storytelling is only beneficial for some of the participants (such as those that identified as visual and interpersonal learners) while other styles of reflection such as writing reflection logs were more applicable to participants who identified as intrapersonal learners (Pashler et al., [21]). According to Harttjar [22], the events that occurred between the late 1800s and the early 1900s set the space for the introduction of OT practice. These events include the industrial revolution, immigration and emigration, the pursuit of better health care services, and the First World War. Looking at OT today, it consists of four core components, namely, the person, environment, occupation, and therapist, and a limitation in any of these components means a limitation in the OT practice [23]. It is essential to identify one's strengths and weaknesses, their predispositions, and their learnings as they practice in order to develop in practice, and this can be attained through reflection. As reflective teaching aids teachers in developing their critical thinking [24], reflection in OT can yield similar results, improving a practitioner's clinical reasoning which is essential for practice. The development of OT practice has influenced the way occupational therapists practice OT today and how they will practice going forward. OT's use of activities and reflection to meet client needs and facilitate competency in health professionals has shaped the way for digital storytelling to be included as a medium of conveying the OT curriculum.

## 7. Situating Digital Storytelling in the OT Curriculum

Reflection and clinical reasoning are seen as an integral part of occupational therapy practice [4, 25], and digital storytelling is a way to combine personal and professional reflection to develop these important facets of practice. Similar to the study conducted by Skarpaas et al. [4], participants in the study agreed that digital storytelling helped them reflect on their experiences and highlighted what they had learnt from the particular practice learning block. They described and saw it as an addition to the other methods of reflection they have used throughout the years and were excited about the addition of something new that could cater and work for the students who are more creatively inclined and not necessarily confident in their written reflections.

### 7.1. Integrating Technology into Education

The global evolution in education that has been driven primarily by a need to keep up with the developed Global North has meant that there has been urgency in the need to integrate technology into education [6]. A participant shared her apprehensiveness about accepting the digital age and immersing herself in technology. E-learning policies within higher education are written with technocratic framing and technological determinism; this combination is said to lead to discourse about the best and latest technology advancement in the curriculum leading to sometimes unclear learning objectives [8]. Sadik [26] speaks about moving classrooms from a space of traditional passive lecture-based teaching and flipping them to an interactive and creative teaching space using technology to engage students more meaningfully with the content, enhancing the students' learning. Participants echoed this statement; they found the use and exposure to a different tool to use for reflection an exciting experience that fed more to their creative- and technology-driven side which facilitated their learning process, and for some, it allowed for deeper reflections into their professional identity. The success of integrating technology into teaching relies on the ability of the chosen technology medium to engage and draw students into wanting to learn [27].

### 7.2. Students' Emotional Impact and the Influence of Context

One of the barriers in the process of engaging with the digital storytelling identified by the participants was the time frame in which the assignment was given. Participants expressed how the external emotional climate influenced how they engaged with the tool. Fourth year participants also expressed how in their third year (which is when the tool was introduced) they felt overwhelmed.

Occupational therapists are very familiar with the notion of the context influencing the person; also, their context can also shape how they engage in certain occupations [28]. It can be safely said that what was going on in and around the students' lives influenced their engagement. Dickie et al. [29] further support this by dissecting the idea of the transactional view, which looks at the concepts of person and environment as a part of each other as opposed to separate from each other. When understanding this in relation to the study, it suggests that the participant's emotional state cannot be separated from the environmental climate. Dickie et.al [29] further explain that in traditional OT literature, person and context are always separated and regarded as separated entities centred on the individual as opposed to looking at the holistic experience of the individual.

The optimal environment to create a digital story was not provided for the participants as described by Grubium [30]. Additionally, fourth year participants shared that the timing of the task was not appropriate as it was given at a time where the faculty was mourning. It affected the engagement of the participants within the process as the task was time-consuming and working with technology proved to be frustrating. Again, this emphasises the assumptions the researchers had prior to conducting the study. The social and emotional environment in which the participants found themselves affected their digital storytelling process and resulted in participants not being able to immerse themselves into the process of creating a digital story but rather complete the task as requested by the lecturers. Similarly, learners from Sadik's [26] study echoed the same sentiments as the participants in this regard.

## 8. Conclusion

This study was aimed at describing UCT occupational therapy student's experiences with using digital storytelling as a reflective tool. Based on the limited literature on digital storytelling within the South African context, the study was aimed at providing more insight into student experiences using digital storytelling as a reflective tool within the OT curriculum. Digital storytelling worked well for students who identified as visual and interpersonal learners. Learners who identified as intrapersonal preferred the written reflective narrative approach. The experience of the process allowed for autonomy in choosing their narrative and creativity within the creation process.

## Data Availability

The qualitative data used to support the findings of this study are included within the article as verbatim quotes. The initial coding data used to support the findings of this study are included within the supplementary information file(s) (available here).

## Supplementary Materials

Supplementary Materials Supplementary Table 1: the initial coding data from the qualitative content analysis.
