# Supplementary material for: Digital Storytelling as a Reflective Tool in Occupational Therapy Curriculum
Source: Occup Ther Int. 2021 Sep 16;2021:2463916. doi: 10.1155/2021/2463916 (PMC8460380; doi:10.1155/2021/2463916)
Supplement: Supplementary Materials — Supplementary Table 1: the initial coding data from the qualitative content analysis. [file 2463916.f1.docx]

Table 1: Data set: Coding Table retrieved from first level analysis

|  | Various perspectives (green) | Process of digital storytelling (purple) | Perceived benefits (Pink) | Perceived barriers (Blue) |
| --- | --- | --- | --- | --- |
| Participant 1 | - “I think its making use of technology...so you can narrate a story” - “I initially wasn't sure how to do it...not exactly sure how to start it.” - “I kinda enjoyed making something rather than writing out an essay.” - “I had to get over that thing of my voice being recorded and having to listen to it again afterwards.” - “I remember just feeling lost” - “I wasn’t interested in it… so I didn’t put much effort into it.” - “Personally I don’t like being recorded … it’s something that I kind of had to adjust to” - “In third year we quite overwhelmed… Takes a lot out of you as well” | - “It was after our first block from physical...by two of the lecturers” - “So we then did a digital story on one of our our block experiences” - “I then had to come up with an idea” - “We tell our story by doing a voice recording ban adding in some slides for a PowerPoint” - “They did have a tutorial kind of lecture...when we first learnt about it.” | - “They gave us a lot of room for interpretation on the topic” - “I kinda enjoyed making something rather than writing out an essay” - “The technical use of like, how to add in recordings to a Powerpoint” - “They did have a tutorial kind of lecture...when we first learnt about it.” - “Good reflecting space...creating something but also creating the opportunity to think back on an incident.” | - “it was quite a stressful time” - “I think it would be challenging … if I didn’t have some experience already with like working with computers” - “They didn’t make that accessible afterwards … he was going to upload it, and he didn’t” - “Probably the time” - “You don’t really have the energy and the motivation to then do something extra” |
| Participant 2 | - “You take an experience or a phenomenon or something, I would say it has to be personal” - relaying it in a way that is a story but through digital means” - “We have to do it we don't have a choice” - “I kind of saw it as just this like added extra thing. - “Experience that I wanted to share with people.” - “ just had a lot of fun doing it.” - “it was a very reflective experience to forcing us to go a little bit deeper into one or more experiences more than you might go into a learners log or a reflective log.” - “It is a nice way to engage with an experience.” - “The process of doing it is very helpful and I will definitely use it with other people.” - “I’ve always been good with technology.” - “No, I don’t want to learn about this.” - It did facilitate my learning experience.” - “It added that fine element that you can play and bring your creativity rather than just typing words on a page.” - (So were you more interested in engaging the task because of the use of technology?) “Yeah i would say so. Definitely.” - “I think I liked it .. very different from anything that I have done before.” - “I absolutely loved my digital storytelling.” - “Because a lot of people they had two amazing mental health blocks where they grew … So maybe just opening it up to more experiences yeah.” - “I really enjoyed.” | - “You take an experience or a phenomenon or something, I would say it had to be personal” - relaying it in a way that is a story but through digital means” - “Told by one of our lecturers they are introducing this new thing called digital storytelling” - “Take an experience from one of our physical health blocks, and used digital storytelling to portray that story and get marked on it” - “Learn more” - “Reflect more” - It forced me to first of all find an experience and then when i read the brief again then an experience immediately came to mind that I wanted to reflect on more” | - “It was a reflective experience.” - “Forcing us to go a little bit deeper into one or more experiences, more than you might go into a learner’s or a reflective log.” - ‘It’s a nice way to engage with an experience.” - “I've always been good with technology” - “You can play and bring your creativity rather just typing words on a page” - (So were you more interested in engaging the task because of the use of technology?) “Definitely” - The actual video that i made and had so much time doing it” - “Working with my own experience that I chose made it enjoyable” - (Did you find any aspects of creating a digital story challenging? )“Not the actual making” - “I have been very fortunate to my school in high school used a lot of technology” - “Digital literacy” - I it as a growing experience, growing not only your skills with technology but then also reflecting in a different way. | - “We have to do it we don’t have a choice.” - “Technology does not go your way.” - “The experience that i chose was not that nice for my client.” - “It just took a long time.” - “I think it’s just understanding what constitutes as an experience to reflect on” - “I don’t understand why it only had to be physical health.” - “Not everyone loves technology like loves making Powerpoint presentations, not many people know how to do a voice over.” - “Not everyone is used to technology in here.” - “Access to that creative technology .” - “Not everyone has a laptop.” |
| Participant 3 | - “I feel like it’s a subjective way of conveying your personal learnings” - “Very creative way of kind of incorporating your theoretical knowledge with also some of the practical experience that you have”. - “I really enjoyed digital storytelling” - “I was actually quite excited, i thought i had a good understanding of what it was” - “I also felt like it was a very subjective experience and process” - “I had a lot of independence on how i got to convey my learnings” - “I think I achieved my objectives even though the mark didn't show” - “I'm gonna make it as creative as I possibly can... but I think that I lacked the creative part” - “I felt like I really on my emerging capabilities as an Occupational Therapist” - “It definitely helped with my learning and solidifying what I had already come to know” - I again liked the creative process behind it...it wasn't as structured as like a typical assignment...you could take it wherever you wanna go” - “It's nice as a per tool but I would have liked it to be kind in a collaborative way within the class as well… you could have learned from someone else's digital storytelling - “I liked the digital aspect of it” - “Its context related...it won't be something that can be a mainstream tool” - “I mean you go to lectures and see slides and that can kind of be tedious” - “So having digital storytelling as a medium of teaching would be exciting for me I’d actually pay more attention.” | - “I feel like it’s a subjective way of conveying your personal learnings” - “Very creative way of kind of incorporating your theoretical knowledge with also some of the practical experience that you have” - “We were given a lecture about it and also just a description about what it is and what was needed from us” - “Convey your reflections on your learnings in your practical blocks...we could use our learners logs as a tool of helpings us...reflect on our learnings” - “I again liked the creative process behind it” - “The objectives maybe weren't clearly defined in terms of what they wanted to see because of the creative element of it” | - “I remember having like some kind of knowledge before” - “I had a lot of independence on how i got to convey my learnings” - “I felt like I really reflected on my emerging capabilities as on occupational Therapist” - (Did the use of a technology medium facilitate your learning experience?) “Definitely” - “it definitely helped with my learning and solidifying what I had already come to know” - (Were you more interested in engaging in the task because of the use of the digital medium?) “Yeah, I was” - “You could really take it wherever you wanna go” - “Just the independence of it...being able to structure it the way you want” - “I was able to do all of that on my own” - “Havin a different medium of being taught, can really spark interest” - “I’m a visual learner” - “So having digital storytelling as a medium of teaching would be exciting for me I’d actually pay more attention.” | - “The objectives maybe weren't clearly defined in terms of what they wanted to see because of the creative element of it...I think that could have been really outlined” - “Access to those digital things...it depends on the kind of the equipment thats available you...internet access” |
| Participant 4 | - “conveying, like a narrative by   using … any media ... a series of pictures) with a certain storyline.”   - “I kind of made it more about myself than an assignment.” - “You really need to think about what kind of pictures you gonna put in your digital story because some pictures might be misinterpreted in like other ways. ” - (did the use of technology a technology medium facilitate your   learning experience?) “Ya sometimes it does facilitate.”   - “I learnt like how much groth i’ve experienced during my block.” - “Using technology is very frustrating.” - “It would give like our lecturers a bit more of a glimpse on what we go through in block.” - “it’s such an emotional taxing   thing to do; to be able to open yourself up to- sometimes lecturers that are insensitive.” | - “Two of our physical health lecturers came … and introduced the whole idea of digital stories and then we had to do an assignment based on our … physical health block. - “the objective was to just tell our experiences of how we   experienced block … and just reflecting upon what we’ve learnt.”  “Building my narrative … like putting it down in words then putting in pictures and then putting in music.” | - “It was like a learning journey for me on how to use it.” - (did the use of technology a technology medium facilitate your   learning experience?) “Ya sometimes it does facilitate.”   - “We’re moving towards ... the fourth industrial revolution, which is more technological. - “Putting it into pictures kind of made it clear for me … my learning journey.” - “It was very interesting for me … it kind of like made it more real … the learning process.” - “It would give like our lecturers a bit more of a glimpse on what we go through in block.” | - “ I think at first it was scary because I’m   not really like a technological person, I’m not really good with like being creative.   - “It was a bit hard to understand it as first. - “It was just a lot of effort. - “You really need to think about what kind of pictures you gonna put in your digital story because some pictures might be misinterpreted in like other ways. ” - “I’m not really technological ... I prefer paper, I like … prefer writing things.” - “I don’t know a lot about technology.” - “I did not like using technology, it was so hard.” - It would be much appreciated if it had more training.” - “It is such an emotional taxing thing to do.” |
| Participant 5 | - “It’s a way for people to learn in a different way and also a way of assessment.” - “Everyone was engaged.” - “If I’m just hearing a voice I can zone out really quickly so it was successful in that way.” | - “We were doing the course, master’s course of OT and primary health care and we were given a task during our lecture week.” - “The purpose of the task was to interview an OT working in the public sector and basically just tell their story of how it is to work in the public sector.” - “having to record the little bits for each slide … then with the whole transitioning from one slide to another.” | - “This is a way to remove like performance anxiety.” - “Incorporate technology … to stay relevant.” - “It can be a learning, like trigger facilitator.” - “This is a way to really prepare something well and then for it to just be executed with no pressure.” - “It made the lecture space more exciting.” - “It definitely made the learning space a lot more engaging, and to have visual cause i like to have visuals.” - “It gives the opportunity for you to not have such stagefright.” - “It just changed the learning space.” - “It helps people to invest in their learning because you are teaching your peers and your peers are teaching you, and that’s how we as adults are supposed to learn. | - “I kinda struggled with it.” - “My recordings were not compatible with that version of powerpoint.” - “I ran out of time.” - “Technology sometimes does not work with us.” - “It was making things more complicated.” - “It was tedious.” - “It was time consuming.” - “It may be a bit too hard considering that we all come in with the different computer skills.” - “It requires a lot of tech skills.” - “Considering time it takes and if people don’t have laptops and access to computers near where they live, that can be a barrier.” |
